# Diagnostic accuracy of point-of-care ultrasound (PoCUS) for the diagnosis of hip effusion in the pediatric emergency department

**DOI:** 10.1007/s43678-024-00788-z

**Published:** 2024-10-01

**Authors:** Hadas Katz-Dana, Rudica Stackievicz, Elad Dana, Nir Friedman, Gali Lackner, Ehud Rosenbloom, Ayelet Shles

**Affiliations:** 1 Pediatric Emergency Department, Meir Medical Center, Kfar Saba, Israel; 2 School of Medicine, Tel Aviv University, Tel Aviv, Israel; 3 Department of Radiology, Intensive Care and Pain Medicine, Meir Medical Center, Kfar Saba, Israel; 4 Department of Anesthesia, Intensive Care and Pain Medicine, Meir Medical Center, Kfar Saba, Israel; 5 Department of Pediatrics, Meir Medical Center, Kfar Saba, Israel

**Keywords:** PoCUS, Ultrasound, Limp, Hip effusion, Septic arthritis, PUCU, Echographie, Boiterie, Effusion de la hanche, Arthrite septique

## Abstract

**Purpose:**

A new limp or refusal to weight-bear are common symptoms in children presenting to the pediatric emergency department (ED). This poses a diagnostic challenge, particularly among toddlers and nonverbal patients. Point-of-care ultrasound (PoCUS) used by pediatric emergency medicine physicians may detect hip effusion, which dramatically aids diagnostic workup and management. There is limited literature regarding the accuracy of hip PoCUS conducted by pediatric emergency medicine physicians. This study aims to assess the diagnostic performance of pediatric emergency medicine physician-performed PoCUS in identifying hip effusion.

**Methods:**

This prospective study was conducted in a single-center pediatric ED. Children presenting with limb pain or new limp were evaluated by pediatric emergency medicine physicians who also performed hip PoCUS and categorized findings as either “effusion” or “no effusion” based on standard sonographic definitions. Patients also underwent radiology department ultrasound reviewed by a pediatric radiologist. Diagnostic test characteristics with corresponding 95% confidence intervals (CI) were calculated using radiology department ultrasound findings as the reference standard.

**Results:**

A total of 95 patients were enrolled by 8 pediatric emergency medicine physicians. Excellent agreement was observed between PoCUS performed by pediatric emergency medicine physicians and radiology department ultrasound for the presence or absence of hip effusion (kappa = 0.81 [95% CI 0.70–0.93]). Hip effusion was identified by PoCUS in 44 out of 49 effusion-positive patients, with a sensitivity of 89.8% (95% CI 77.7–96.6%), specificity of 91.3% (95% CI 79.2%-97.5%), positive likelihood ratio of 10.33 (95% CI 4.03–26.47), and negative likelihood ratio of 0.11 (95% CI 0.05–0.26).

**Conclusion:**

PoCUS performed by pediatric emergency medicine physicians has reasonably high sensitivity and specificity for diagnosing hip effusion among pediatric patients presenting to the pediatric ED with a limp or leg pain. This practice may potentially expedite both diagnosis and treatment within this patient population.

## Résumé

**Objectif:**

Un nouveau boiteux ou un refus de porter le poids sont des symptômes courants chez les enfants qui se présentent à l’urgence pédiatrique (DE). Cela pose un défi diagnostique, en particulier chez les enfants en bas âge et les patients non verbaux. Les échographies de point de soins (PUCU) utilisées par les médecins des urgences pédiatriques peuvent détecter un épanchement de la hanche, ce qui facilite considérablement le diagnostic et la gestion. Il existe une littérature limitée concernant la précision des PUC de la hanche effectuée par les médecins urgentistes pédiatriques. Cette étude vise à évaluer la performance diagnostique des PUCU réalisées par un médecin en médecine d’urgence pédiatrique pour identifier l’effusion de la hanche.

**Méthodes:**

Cette étude prospective a été menée dans un seul centre de DE pédiatrique. Les enfants présentant une douleur aux membres ou une nouvelle boiterie ont été évalués par des médecins pédiatriques d’urgence qui ont également effectué un PUCU de la hanche et ont classé les résultats comme "épanchement" ou "aucun épanchement" selon les définitions échographiques standard. Les patients ont également subi une échographie du service de radiologie examinée par un radiologue pédiatrique. Les caractéristiques des tests diagnostiques avec leurs intervalles de confiance (IC) correspondants à 95 % ont été calculées en utilisant les résultats d’échographie du service de radiologie comme norme de référence.

**Résultats:**

Un total de 95 patients a été inscrits par huit médecins urgentistes pédiatriques. Une excellente concordance a été observée entre les ultrasons réalisés par les médecins pédiatriques d’urgence et ceux du service de radiologie pour la présence ou l’absence d’effusion de la hanche (kappa = 0.81 [IC à 95% 0.70–0.93]). Le épanchement de la hanche a été identifié par PUCU chez 44 des 49 patients ayant un épanchement positif, avec une sensibilité de 89,8 % (IC à 95%, 77.7 –96.6 %), une spécificité de 91,3 % (IC à 95%, 79.2–97.5%), un rapport de vraisemblance positif de 10,33 (IC à 95 %, 4.03–26.47) et un rapport de vraisemblance négatif de 0,11 (IC à 95% 0.05-0.26)

**Conclusions:**

Le PUCU réalisé par des médecins pédiatriques d’urgence a une sensibilité et une spécificité raisonnablement élevées pour diagnostiquer l’épanchement de la hanche chez les patients pédiatriques présentant une lésion ou une douleur aux jambes. Cette pratique pourrait accélérer le diagnostic et le traitement dans cette population de patients.

## Clinician’s capsule

**Table:** 

***What is known about the topic?***
Pediatric patients presenting with leg pain or a new limp are increasingly assessed using ultrasound performed by pediatric emergency physicians.
***What did this study ask?***
What is the diagnostic accuracy of hip point of care ultrasound performed by pediatric emergency physicians compared to radiology ultrasound?
***What did this study find?***
Point of care ultrasound had approximately 90% sensitivity and specificity for identifying hip effusion.
***Why does this study matter to clinicians?***
Accurate hip effusion detection by emergency physicians at the bedside may improve diagnostic efficiency and reduce the use of radiology resources.

## Introduction

Limping, lower limb pain, or refusal to weight-bear are common symptoms among patients presenting to the pediatric emergency department (ED) [1]. These complaints stem from various causes, ranging from benign and self-limiting conditions like transient synovitis to critical surgical emergencies like septic arthritis. To correctly diagnose the etiology and guide specific management, clinicians often employ additional diagnostic tests, with ultrasound being the most common modality.

Hip ultrasonography is traditionally conducted primarily by radiologists [2]. However, even in as early as 1999, Smith published a case report demonstrating the potential of point-of-care ultrasound (PoCUS) for diagnosing hip effusion and guiding arthrocentesis in an adult patient [9]. The first larger prospective study on PoCUS for hip effusion was published in 2010, further supporting PoCUS use in this context [10]. Recently, the use of PoCUS by pediatric emergency medicine physicians has become an invaluable tool for patient assessment [5–11]. This non-invasive diagnostic tool contributes to a more accurate diagnosis while reducing complications and decreasing length of stay [12–14].

Despite the growing use of hip PoCUS in evaluating children with a limp or limb pain, a comprehensive review of the literature reveals a dearth of data regarding the accuracy of hip PoCUS when performed by pediatric emergency medicine physicians. We aimed to determine the diagnostic accuracy of hip PoCUS conducted by pediatric emergency medicine physicians compared with the ultrasound scan performed by radiologists.

## Methods

### Study design, setting, and time period

This is a single-center, prospective observational study, conducted at the pediatric ED of Meir Medical Center, an academic hospital that sees approximately 400,000 children annually. Between October 1st, 2020, and September 15th, 2021, children presenting with limping, lower limb pain, or refusal to bear weight underwent PoCUS assessment by pediatric emergency medicine physicians, followed by a radiologists’ scan reviewed by a pediatric radiologist (reference standard). Scan results were compared to assess the diagnostic accuracy of PoCUS performed by pediatric emergency medicine physicians vs. the reference standard.

Bedside hip PoCUS was performed by five pediatric emergency medicine attending physicians and three pediatric emergency medicine fellows with at least 2 years of experience in PoCUS following a short training program provided by an expert PoCUS pediatric emergency medicine physician. This training comprised a 2-h didactic session covering fundamental ultrasound principles and the sonographic diagnosis of hip effusion, followed by a 2-h hands-on session in which all sonographers performed six hip scans on live models under supervision. Physicians were then required to submit ten hip PoCUS scans for evaluation, including at least one positive scan (all PoCUS scans were recorded on a Zonare One Pro device by Mindray, Shenzhen, China). Following this three-step process, physicians were credentialed to perform hip PoCUS and participate in the study.

The study was approved by the Meir Medical Center institution ethical committee for medical and health research ethics (MMC nr. 0119–20) in accordance with the declaration of Helsinki.

## Population

Patients were eligible to participate if they presented to the pediatric ED with symptoms of a new limp, lower limb pain, or refusal to bear weight, with hip ultrasonography required as part of their assessment, and ordered by a pediatric emergency medicine physician certified to perform hip PoCUS following the training program. Patients were enrolled according to the availability of the study physicians. Patients with a history of diagnostic hip imaging within the week prior to their evaluation in our ED were excluded.

A convenience sample was used due to the availability of scanning pediatric emergency medicine physicians on site. Patient enrollment was contingent upon the availability of study physicians. Written informed consent was obtained from legal guardians of all patients prior to enrollment.

## Intervention

**Fig. 1:**
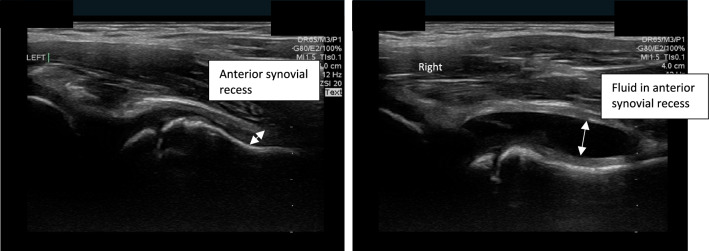
A Normal hip PoCUS. B Hip PoCUS demonstrating a joint effusion. There is an anechoic fluid collection between the anterior surface of the femoral neck and the posterior surface of the iliopsoas muscle (white arrow)

After informed consent was obtained, study physicians conducted bedside PoCUS on both the affected hip as well as the contralateral hip of each patient. Patients were positioned in the supine position, with the examined hip joint slightly abducted and externally rotated. The linear probe was oriented parallel to the femoral neck in the sagittal plane. The presence or absence of hip effusion was determined by identifying the synovial space and measuring the distance between the anterior surface of the femoral neck and the posterior surface of the iliopsoas muscle for both legs. A measurement greater than 5-mm or greater than a 2-mm difference from the contralateral hip was considered positive for hip effusion [15, 16]. Figure 1 offers visual representations of a normal hip (A) and a hip with effusion (B). PoCUS scans were recorded and stored on the PoCUS device. For every scan, the study physicians interpreted their measurements as either “effusion” or “no effusion,” based on the provided definition. Pediatric emergency medicine PoCUS providers were not blinded to patients’ clinical information. Radiology ultrasound scans were performed within 2 h of the PoCUS scan (median time 95 min) by radiologists, who were blinded to the pediatric emergency medicine PoCUS results. These scans were recorded on their device, to also be reviewed by a staff pediatric radiologist within 24 h following patients’ presentation to the pediatric ED. The pediatric radiologist’s interpretation of scans served as the reference standard. Outcomes were documented on standardized data sheets. Following the radiology ultrasound scan, further diagnostic procedures (blood work, additional imaging, and arthrocentesis) and treatment were considered according to our institutional protocol. In cases where arthrocentesis was indicated, it was usually performed within 1 h in the imaging department by an orthopedic surgeon under real-time ultrasound guidance.

## Data analysis

Standard methods were used to provide tabular and graphical summaries as appropriate for continuous and categorical variables. The Shapiro–Wilk test was used to assess the normality of the data distribution. Continuous variables are presented as mean ± standard deviation (SD) if normally distributed, otherwise as medians [interquartile ranges]. Categorical variables are expressed as absolute numbers (%). For our primary outcome, the diagnostic agreement between the PoCUS and radiology department US scans, Cohen’s Kappa value was calculated [17]. A kappa coefficient of over 0.75 is considered indicative of near-perfect agreement [18]. PoCUS predictive values were computed in relation to radiology department US results, along with corresponding 95% confidence intervals (CI). All statistical analyses were conducted using SPSS (version 20; SPSS Inc., USA). Statistical significance was set at *p* < 0.05.

## Results

**Fig. 2:**
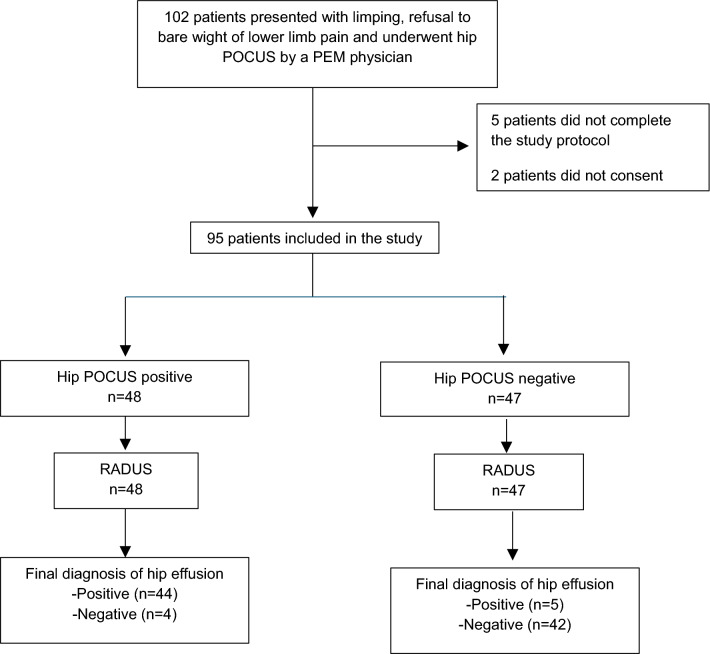
Study flowchart

During the study period, 102 patients presented with symptoms of limping, lower limb pain, or refusal to bear weight and underwent PoCUS by pediatric emergency medicine physicians as part of their clinical workup. Of these patients, five (5%) did not complete the study protocol and two (2%) did not consent to participate in the study. Thus, after obtaining written informed consent, 95 patients were included in the study. Figure 2 depicts the study flow diagram. All patients’ data were obtained, and all patients were included in our final analyses. Baseline demographic and clinical characteristics of included patients are presented in Table 1.

**Table 1:** Baseline demographic and clinical characteristics

Variable	Patients (*n* = 95)
Age, mean (SD), years	6.4 (3.1)
Female sex, n (%)	28 (29.5)
Clinical parameters	
Fever, *n* (%)	16 (16.8)
Elevated CRP, *n* (%)*	19 (54.3)
Elevated WBC, *n* (%)*	3 (9.1)
Admissions (%)	14 (14.7)
Diagnosis	
Transient synovitis (%)	76 (80)
Septic arthritis (%)	3 (3.1)
Other (%)	16 (16.9)

^*^% refers to valid values of children who underwent blood work

**Fig. 3:**
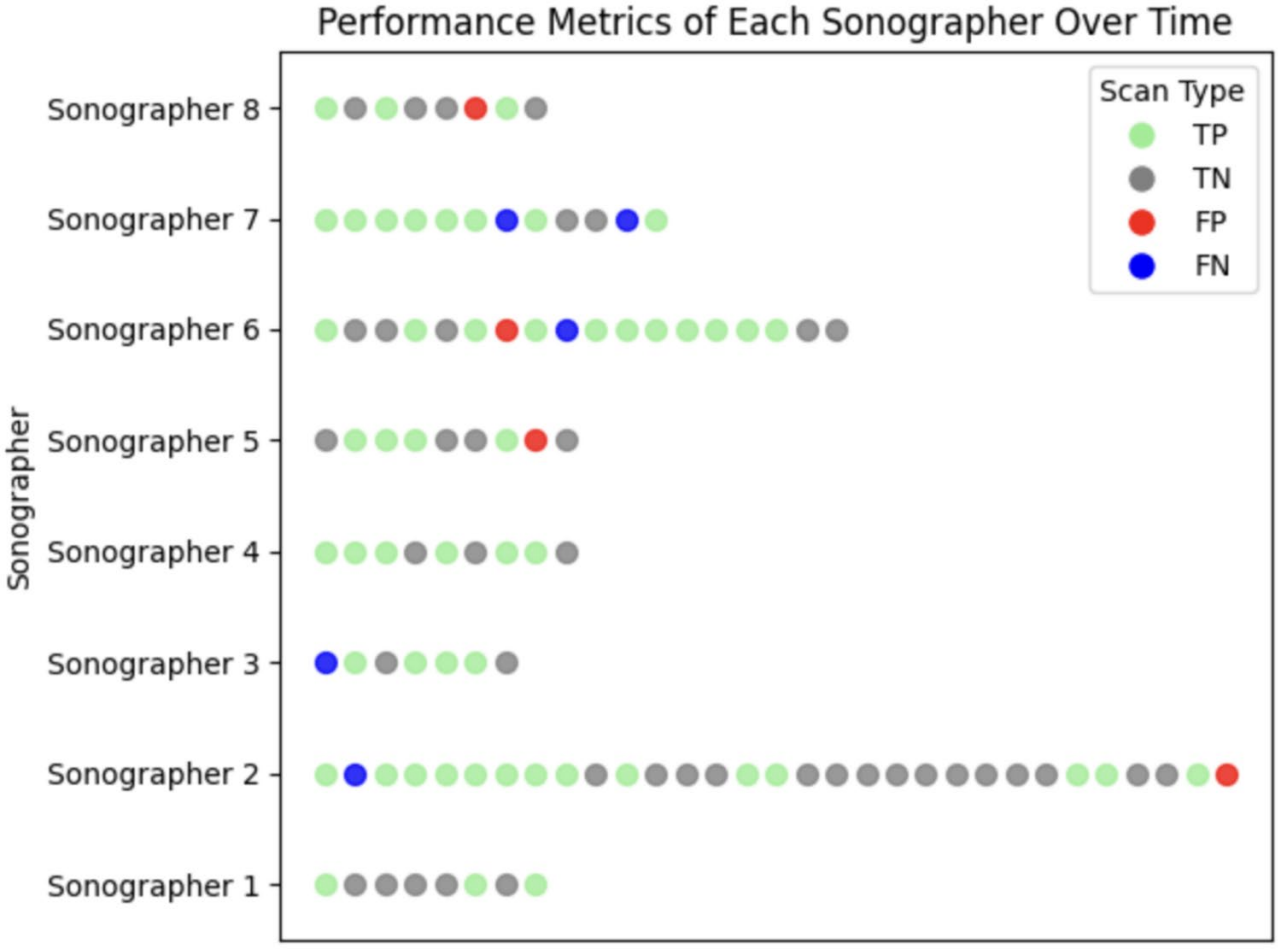
Performance metrics of each sonographer over time. Sonographers are labeled on the y-axis, while time is represented on the x-axis. Each circle represents a scan, color-coded to indicate its type: green for true positives (TP), grey for true negatives (TN), pink for false positives (FP), and blue for false negatives (FN). The legend clarifies the scan types. This visualization provides an overview of sonographer performance trends

There was an excellent agreement between the PoCUS and radiology ultrasound results (kappa = 0.81, 95% confidence interval [CI] 0.70–0.93). PoCUS performed by pediatric emergency medicine physicians had a sensitivity of 89.8% (95% CI 77.7–96.6%), specificity of 91.3% (95% CI 79.2–97.5%), positive predictive value (PPV) of 91.5% (95% CI 81.1–96.5%), and a negative predictive value (NPV) of 89.6% (95% CI 85.05–93.74%). The positive likelihood ratio was 10.33 (95% CI: 4.03–26.47), and the negative likelihood ratio was 0.11 (95% CI: 0.05–0.26). Table 2 describes the diagnostic test characteristics of PoCUS for hip effusion. Figure 3 outlines the results of each PoCUS scan in the order it was performed by each pediatric emergency medicine physician to provide an overview of sonographer performance trends.

**Table 2:** Comparison of accuracy of PEM-performed PoCUS vs. RADUS diagnoses

	RADUS		
	Positive, n	Negative, *n*	Row total, *n*
PoCUS			
Positive, *n*	44	4	48
Negative, *n*	5	42	47
Column total, *n*	49	46	

The calculated performance parameters of PoCUS performed by PEM physicians compared to RADUS are: sensitivity 89.8% (95% CI 77.7–96.6), specificity 91.3% (95% CI 79.2–97.5); PPV 91.5% (95% CI 81.1–96.5), NPV 89.6% (95% CI 78.4–95); + LR 10.33 (95% CI 4–26.5),  – LR 0.11 (95% CI 0.05–0.26)

*PEM* pediatric emergency physician, *RADUS* radiology department ultrasound, *PoCUS* point-of-care ultrasound, + *LR* positive likelihood ratio, – *LR* negative likelihood ratio

Diagnostic disagreement between PoCUS and radiology ultrasound studies occurred in nine (9.4%) cases—four (4.2%) false positives and five (5.2%) false negatives. All false negative results and three of the false positive results occurred due to incorrect measurement of the joint capsule, leading to either failure to identify an existing effusion (false negative) or overestimating the capsule space, misinterpreted as the presence of an effusion (false positive). In one case, the study physician misidentified a tendon for an effusion due to under gaining of the ultrasound image, leading to a false positive result. No difference was observed in the PoCUS/radiology department US disagreement rate when PoCUS was performed by a pediatric emergency medicine fellow or staff physician (*p* = 0.27).

Among the 14 (14.7%) patients that were eventually admitted, there was perfect agreement (kappa = 1) between PoCUS and radiology ultrasound results regarding the presence of hip effusion. Within this group, 3 patients had a final diagnosis of septic arthritis and 11 had transient synovitis. Of patients with septic arthritis, two had a hip effusion that was detected by both PoCUS and radiology ultrasound providers. The third patient was negative for hip effusion by both PoCUS and radiology ultrasound.

## Discussion

### Interpretation of findings

This study, the largest prospective investigation assessing the diagnostic performance of hip PoCUS performed by pediatric emergency medicine physicians to date, demonstrates that this tool is reliable for detecting hip effusion among pediatric patients presenting to the pediatric ED with symptoms of limping or leg pain. The agreement between pediatric emergency medicine-performed PoCUS and radiology ultrasound was either excellent [18] or almost perfect [19]. A perfect (Kappa value = 1) agreement was observed when focusing on all 14 patients who required admission, and more importantly when focusing on the 3 cases of septic arthritis, a not-to-be-missed emergency. This highlights the potential of PoCUS in enabling timely intervention for critical conditions. It should be emphasized that in our study, PoCUS mistakenly identified a hip effusion in four patients (false positive) and failed to detect an effusion in five (false negative). As previously described in other studies, the inaccurate measurement of sonographic landmarks within the synovial space was the primary cause [10]. Overall, false positive and false negative rates were low. We emphasize that the clinical management in these cases does not solely rely on the sonographic presence of a hip effusion but takes other clinical factors into consideration.

### Comparison to previous studies

Our findings are concordant with previous reports on the benefits of PoCUS in the diagnosis and expedited care of pediatric patients presenting with limping or refusal to weight-bear [3, 20–22]. In a retrospective report by Cruz et al. [21], which included 516 patients, the sensitivity and specificity of hip ultrasound for diagnosing hip effusion were similar to those in our study, at 85% and 98%, respectively. In a prospective study by Vieira et al. [10], which included 28 pediatric patients, PoCUS performed by pediatric emergency medicine physicians had a sensitivity of 80% (95% CI 51–95%) and specificity of 98% (95% CI 85–99%). Studies within the pediatric ED setting also demonstrated the ability of PoCUS to reduce unnecessary diagnostic procedures, and shorten emergency department stays [23, 24]. In a recent small-scale case series [22], Berant and colleagues showcased the safety and feasibility of PoCUS-guided hip aspiration in diagnosing septic hip cases within the pediatric ED [22]. These outcomes substantiate the integration of hip PoCUS, into the realm of emergency physicians, enhancing diagnostic accuracy and expediting the care of pediatric patients with musculoskeletal complaints. While bedside ultrasonography is being integrated into emergency medicine residency and pediatric emergency medicine fellowship curricula, there remains a lack of consensus regarding the inclusion of hip PoCUS application in PoCUS training programs [25]. In our study, as well as in existing literature, only brief training was required to equip pediatric emergency medicine physicians with hip PoCUS scanning capability. We conducted a 2-h didactic session, 2-h hands-on training, and additional 10 live scans on actual patients. In a study by Pade Et Al, a brief training program for novice PoCUS users to accurately identify hip anatomy and hip effusion had excellent results [26]. We hypothesize that, as evidence of the accuracy and implications of this practice accumulate, more consistent and high-quality training programs will be integrated into emergency care curriculum.

### Strengths and limitations

This study has several strengths. First, it is the largest prospective investigation specifically assessing the diagnostic performance of hip PoCUS performed by pediatric emergency medicine physicians in pediatric patients to date. The comprehensive nature of our study, encompassing a substantial number of cases, enhances the reliability and generalizability of the observed results. Second, the utilization of Cohen’s Kappa value in assessing agreement adds a quantitative dimension to the evaluation, providing a standardized measure of reliability. These strengths collectively contribute to the significance of our study.

There are several limitations to our study. First, it relied on convenience sampling, which may introduce selection bias and limit generalizability of findings. Furthermore, as the study lacked a sample size or power calculation, the precision and confidence in the observed results are limited. Second, our study was conducted in a single-center pediatric ED with an established PoCUS training program. The feasibility of implementing hip PoCUS in centers lacking such resources remains uncertain. Third, the non-blinded nature of the study, with scanning pediatric emergency medicine physicians potentially incorporating clinical information into their image interpretation and decision-making process, mirrors real-world scenarios but introduces potential bias. Finally, the study’s limited number of participating pediatric emergency medicine physicians raises concerns about generalizability, given the operator-dependent nature of ultrasonography.

### Clinical implications

Our findings advocate for the incorporation of hip PoCUS into the diagnostic approach for pediatric patients presenting with limping, hip pain, or refusal to weight-bear at the pediatric ED. The ability of pediatric emergency medicine physicians to rapidly and accurately identify hip effusion, particularly in critical conditions like septic arthritis, has substantial clinical implications. It is crucial to note that while a negative PoCUS result is valuable, it does not definitively rule out the possibility of septic arthritis, especially in patients with a high pre-test probability. Therefore, clinical judgment, meticulous assessment of risk factors, and incorporation of additional diagnostic modalities remain paramount in managing patients with suspected hip effusion and concern for septic arthritis. However, incorporating PoCUS in such scenarios not only streamlines diagnostic processes but also enhances the overall quality of care by minimizing the need for unnecessary tests and expediting appropriate evaluation and treatment.

### Research implications

The results of our study suggest promising implications for integrating hip PoCUS into emergency medicine training. However, it is crucial to validate these findings with a consecutive sample that includes a larger and heterogeneous group of sonographers to ensure generalizability. In addition, there is a need for clarity regarding the optimal amount of training required for physicians to proficiently perform hip PoCUS. Future research should also focus on assessing how incorporating hip PoCUS impacts patient-oriented outcomes such as ED length of stay. Moreover, clinicians must be equipped with evidence-based strategies to interpret these findings effectively, considering the pre-test probability of conditions like septic arthritis.

## Conclusion

Pediatric emergency medicine physicians may accurately identify a hip effusion using PoCUS with high sensitivity and specificity compared to a reference standard of radiology ultrasound. Large, randomized trials are required to assess whether this skill may be acquired with reasonable resource expenditure and to determine the impact of hip PoCUS on other patient outcomes, such as time to diagnosis and ED length of stay.

## References

[1] Fischer SU, Beattie TF. The limping child: epidemiology, assessment and outcome. J Bone Joint Surg Br. 1999;81-B(6):1029–34. 10.1302/0301-620x.81b6.9607 10615981

[2] Wilson DJ, Green DJ, MacLarnon JC. Arthrosonography of the painful hip. Clin Radiol. 1984;35(1):17–9. 6690175 10.1016/s0009-9260(84)80219-6

[3] Smith SW. Emergency physician-performed ultrasonography-guided hip arthrocentesis. Acad Emerg Med. 1999;6(1):84–6. 9928982 10.1111/j.1553-2712.1999.tb00101.x

[4] Vieira RL, Levy JA. Bedside ultrasonography to identify hip effusions in pediatric patients. Ann Emerg Med. 2010;55(3):284–9. 19695738 10.1016/j.annemergmed.2009.06.527

[5] Lewis D, Rang L, Kim D, Robichaud L, Kwan C, Pham C, et al. Recommendations for the use of point-of-care ultrasound (POCUS) by emergency physicians in Canada. CJEM. 2019;21(6):721–6. 31771691 10.1017/cem.2019.392

[6] Harel-Sterling M, Diallo M, Santhirakumaran S, Maxim T, Tessaro M. Emergency department resource use in pediatric pneumonia: point-of-care lung ultrasonography versus chest radiography. J Ultrasound Med. 2019;38(2):407–14. 30027608 10.1002/jum.14703

[7] Beyer A, Lam V, Fagel B, Dong S, Hebert C, Wallace C, et al. Undifferentiated dyspnea with point-of-care ultrasound, primary emergency physician compared with a dedicated emergency department ultrasound team. J Emerg Med. 2021;61(3):278–92. 34348868 10.1016/j.jemermed.2021.03.003 PMC8578047

[8] Levy JA, Noble VE. Bedside ultrasound in pediatric emergency medicine. Pediatrics. 2008;121(5):e1404–12. 18450883 10.1542/peds.2007-1816

[9] Marin JR, Abo AM, Arroyo AC, Doniger SJ, Fischer JW, Rempell R, et al. Pediatric emergency medicine point-of-care ultrasound: summary of the evidence. Crit Ultrasound J. 2016;8(1):16. 27812885 10.1186/s13089-016-0049-5 PMC5095098

[10] Tsou P-Y, Wang Y-H, Ma Y-K, Deanehan JK, Gillon J, Chou EH, et al. Accuracy of point-of-care ultrasound and radiology-performed ultrasound for intussusception: a systematic review and meta-analysis. Am J Emerg Med. 2019;37(9):1760–9. 31182360 10.1016/j.ajem.2019.06.006

[11] Katz-Dana H, Harel-Sterling M, Vincent D, Dana E, Navarro OM, McLean LJ. A POCUS-first pathway to streamline care for children with suspected ileocolic intussusception. CJEM. 2024;26(4):235–43. 38538954 10.1007/s43678-024-00673-9

[12] Andersen CA, Holden S, Vela J, Rathleff MS, Jensen MB. Point-of-care ultrasound in general practice: a systematic review. Ann Fam Med. 2019;17(1):61–9. 30670398 10.1370/afm.2330 PMC6342599

[13] Bortcosh W, Shaahinfar A, Sojar S, Klig JE. New directions in point-of-care ultrasound at the crossroads of paediatric emergency and critical care. Curr Opin Pediatr. 2018;30(3):350–8. 29528889 10.1097/MOP.0000000000000621

[14] Chen K-C, Lin AC-M, Chong C-F, Wang T-L. An overview of point-of-care ultrasound for soft tissue and musculoskeletal applications in the emergency department. J Intensive Care. 2016;4(1):55. 27529031 10.1186/s40560-016-0173-0 PMC4983782

[15] Siegel MJ. Pediatric sonography. J Diagn Med Sonogr. 1996;12(4):202–6–202–6.

[16] Cruz CI. Point-of-care hip ultrasound in a pediatric emergency department. Am J Emerg Med. 2018. 10.1016/j.ajem.2017.11.059. 29223689 10.1016/j.ajem.2017.11.059

[17] Landis JR, Koch GG. The measurement of observer agreement for categorical data. Biometrics. 1977;33(1):159–74. 843571

[18] Fleiss JL. Statistical methods for rates and proportions. Newy York: Wiley-Interscience; 1973. p. 248.

[19] McHugh ML. Interrater reliability: the kappa statistic. Biochem Med (Zagreb). 2012;22(3):276–82. 23092060 PMC3900052

[20] Singer JI. The cause of gait disturbance in 425 pediatric patients. Pediatr Emerg Care. 1985;1(1):7–10. 3843430 10.1097/00006565-198503000-00003

[21] Cruz CI, Vieira RL, Mannix RC, Monuteaux MC, Levy JA. Point-of-care hip ultrasound in a pediatric emergency department. Am J Emerg Med. 2018;36(7):1174–7. 29223689 10.1016/j.ajem.2017.11.059

[22] Berant R, Bder M, Kaufman-Shriqui V, Shahar-Nissan K. Point-of-care ultrasound-guided aspiration of the hip joint by an emergency medicine physician. Pediatr Emerg Care. 2022;38(3):139–42. 35226624 10.1097/PEC.0000000000002650

[23] Shavit I. Sonography of the hip joint by the emergency physician. Pediatr Emerg Care. 2006. 10.1097/01.pec.0000230705.51151.40. 16912625 10.1097/01.pec.0000230705.51151.40

[24] Tsung JW, Blaivas M. Emergency department diagnosis of pediatric hip effusion and guided arthrocentesis using point-of-care ultrasound. J Emerg Med. 2008;35(4):393–9. 18403170 10.1016/j.jemermed.2007.10.054

[25] Shefrin AE, Warkentine F, Constantine E, Toney A, Uya A, Doniger SJ, et al. Consensus core point-of-care ultrasound applications for pediatric emergency medicine training. AEM Educ Train. 2019;3(3):251–8. 31360818 10.1002/aet2.10332 PMC6637013

[26] Pade KH, Niknam KR, Lobo VE, Anderson KL. The efficacy of a brief educational training session in point-of-care pediatric hip ultrasound. Pediatr Emerg Care. 2022;38(1):1–3. 32796351 10.1097/PEC.0000000000002202

